# Complete Genome Sequence of the Bacteriochlorophyll *b*-Producing Photosynthetic Bacterium *Blastochloris viridis*

**DOI:** 10.1128/genomeA.01006-15

**Published:** 2015-09-03

**Authors:** Yusuke Tsukatani, Yuu Hirose, Jiro Harada, Naomi Misawa, Keita Mori, Kazuhito Inoue, Hitoshi Tamiaki

**Affiliations:** a Earth-Life Science Institute, Tokyo Institute of Technology, Tokyo, Japan; b PRESTO, Japan Science and Technology Agency, Saitama, Japan; c Department of Environmental and Life Sciences, Toyohashi University of Technology, Aichi, Japan; d Electronics Inspired-Interdisciplinary Research Institute (EIIRIS), Toyohashi University of Technology, Aichi, Japan; e Department of Medical Biochemistry, Kurume University School of Medicine, Fukuoka, Japan; f Department of Biological Sciences, Kanagawa University, Kanagawa, Japan; g Graduate School of Life Sciences, Ritsumeikan University, Shiga, Japan

## Abstract

We report the complete genome sequence of the purple photosynthetic bacterium *Blastochloris viridis* belonging to α-*Proteobacteria*. This is the first completed genome sequence of a phototroph producing bacteriochlorophyll *b*. The genome information will be useful for further analysis of the photosynthetic energy conversion system and bacteriochlorophyll pigment biosynthesis.

## GENOME ANNOUNCEMENT

*Blastochloris viridis* is a member of anoxygenic phototrophic bacteria in the phylum *Proteobacteria* (α-2 subclass) (1, 2), which are often called “purple bacteria.” This bacterium is unique because it produces bacteriochlorophyll (BChl) *b* which has an absorption maximum in the near-infrared light region (3, 4), whereas most isolated purple bacteria produce BChl *a* (5, 6). Photochemical reaction center complexes of *B. viridis* were the target proteins for the study revealing the first crystal structure of membrane protein complexes (7), and three of the authors won the Nobel Prize in Chemistry in 1988. In 2004, we sequenced the photosynthetic gene cluster of *B. viridis* using an inverse PCR and Sanger method, and some genes in the cluster have already been deposited at GenBank (accession no. AB738834). For the better understanding of photosynthetic processes based on BChl *b* and the biosynthetic pathways of the pigment, we determined the complete genome sequence of *B. viridis* DSM 133 (1).

Genome sequencing was performed using the MiSeq system (Illumina). An 800-bp paired-end library and an 8-kbp mate-pair library were prepared using the TruSeq DNA PCR-free sample preparation kit (Illumina) and Nextera mate-pair sample preparation kit (Illumina), respectively. The libraries were sequenced with the MiSeq Reagent kit v3 (600-cycles; Illumina). The reads were filtered using ShortReadManager based on 17-mer frequency (8). A total of 198-Mbp paired-end reads and 218-Mbp mate-pair reads were assembled using Newbler version 2.9 (Roche), yielding 1 scaffold and 58 large contigs (>1 kbp). Gap sequences between the contigs were determined *in silico* using GenoFinisher and AceFileViewer (8), followed by PCR and Sanger sequencing. We succeeded in determining the complete genome sequence of *B. viridis* strain DSM 133, which comprises one circular chromosome of 3,861,362 bp. The G+C content of the genome was calculated to be 67%. Gene prediction and functional annotation were carried out with Rapid Annotations using Subsystems Technology (RAST) (9), revealing 3,576 protein-coding genes, 9 rRNAs, and 47 tRNAs.

Most of the genes related to photosynthetic activities were clustered in a region on the genome forming the so-called photosynthetic gene cluster (PGC) (10). The PGC of *B. viridis* includes genes for photochemical reaction center complexes (*pufLMC* and *puhH*), light-harvesting proteins (*pufBA*), bacteriochlorophyll biosynthesis (*bchPGFNBHLMIDCXYZ*), and carotenoid biosynthesis (*crtIBE*). A characteristic of the PGC of this organism is the presence of genes for carbon fixation in the middle of PGC. The *bchE* and *bchJ* genes are known to be involved in bacteriochlorophyll biosynthesis (11, 12), but are not located in PGC. As we already reported using the draft genome sequence (4), the *bciA* gene encoding divinyl-chlorophyllide reductase found in BChl *a*-producing purple bacteria are missing in the genome of *B. viridis*. This genome information will contribute to our understanding of bacteriochlorophyll biosynthetic pathways and photosynthetic apparatuses in *B. viridis*.

### Nucleotide sequence accession number. 

The complete genome sequence of *B. viridis* DSM 133 has been deposited at the DNA Data Bank of Japan (DDBJ) under accession number AP014854.

## References

[1] Drews G, Giesbrecht P 1966 *Rhodopseudomonas viridis*, nov. spec., ein neu isoliertes, obligat phototrophes Bakterium. Arch Microbiol 53:255–262. 5991629

[2] Hiraishi A 1997 Transfer of the bacteriochlorophyll *b*-containing phototrophic bacteria *Rhodopseudomonas viridis* and *Rhodopseudomonas sulfoviridis* to the genus *Blastochloris* gen. nov. Int J Syst Bacteriol 47:217–219. doi:10.1099/00207713-47-1-217. 8995826

[3] Jay F, Lambillotte M, Stark W, Mühlethaler K 1984 The preparation and characterisation of native photoreceptor units from the thylakoids of *Rhodopseudomonas viridis*. EMBO J 3:773–776. 16453514 10.1002/j.1460-2075.1984.tb01883.x PMC557425

[4] Tsukatani Y, Yamamoto H, Harada J, Yoshitomi T, Nomata J, Kasahara M, Mizoguchi T, Fujita Y, Tamiaki H 2013 An unexpectedly branched biosynthetic pathway for bacteriochlorophyll *b* capable of absorbing near-infrared light. Sci Rep 3:1217. doi:10.1038/srep01217. 23386973 PMC3564038

[5] Stomp M, Huisman J, Stal LJ, Matthijs HCP 2007 Colorful niches of phototrophic microorganisms shaped by vibrations of the water molecule. ISME J 1:271–282. doi:10.1038/ismej.2007.59. 18043638

[6] Mizoguchi T, Harada J, Tamiaki H 2006 Structural determination of dihydro- and tetrahydrogeranylgeranyl groups at the 17-propionate of bacteriochlorophylls-*a*. FEBS Lett 580:6644–6648. doi:10.1016/j.febslet.2006.11.020. 17123518

[7] Deisenhofer J, Epp O, Miki K, Huber R, Michel H 1985 Structure of the protein subunits in the photosynthetic reaction centre of *Rhodopseudomonas viridis* at 3Å resolution. Nature 318:618–624. doi:10.1038/318618a0. 22439175

[8] Ohtsubo Y, Maruyama F, Mitsui H, Nagata Y, Tsuda M 2012 Complete genome sequence of *Acidovorax* sp. strain kks102, a polychlorinated-biphenyl degrader. J Bacteriol 194:6970–6971. doi:10.1128/JB.01848-12. 23209225 PMC3510582

[9] Overbeek R, Olson R, Pusch GD, Olsen GJ, Davis JJ, Disz T, Edwards RA, Gerdes S, Parrello B, Shukla M, Vonstein V, Wattam AR, Xia F, Stevens R 2014 The SEED and the Rapid Annotation of microbial genomes using Subsystems Technology (RAST). Nucleic Acids Res 42:D206–D214. doi:10.1093/nar/gkt1226. 24293654 PMC3965101

[10] Alberti M, Burke DH, Hearst JE 1995 Structure and sequence of the photosynthesis gene cluster, p 1083–1106. *In* Blankenship RE, Madigan MT, Bauer CE (ed), Anoxygenic photosynthetic bacteria. Kluwer Publishers, Dordrecht, The Netherlands.

[11] Chew AGM, Bryant DA 2007 Chlorophyll biosynthesis in bacteria: the origins of structural and functional diversity. Annu Rev Microbiol 61:113–129. doi:10.1146/annurev.micro.61.080706.093242. 17506685

[12] Tsukatani Y, Harada J, Nomata J, Yamamoto H, Fujita Y, Mizoguchi T, Tamiaki H 2015 *Rhodobacter sphaeroides* mutants overexpressing chlorophyllide *a* oxidoreductase of *Blastochloris viridis* elucidate functions of enzymes in late bacteriochlorophyll biosynthetic pathways. Sci Rep 5:9741. doi:10.1038/srep09741. 25978726 PMC4432870

